# Endovascular treatment of aneurysms of the paraophthalmic segment of the internal carotid artery: Current status

**DOI:** 10.3389/fneur.2022.913704

**Published:** 2022-09-16

**Authors:** Yiheng Wang, Jinlu Yu

**Affiliations:** Department of Neurosurgery, First Hospital of Jilin University, Changchun, China

**Keywords:** paraophthalmic segment, internal carotid artery, endovascular treatment, aneurysm, review

## Abstract

The paraophthalmic segment of the internal carotid artery (ICA) originates from the distal border of the cavernous ICA and terminates at the posterior communicating artery. Aneurysms arising from the paraophthalmic segment represent ~5–10% of intradural aneurysms. Due to the advent of endovascular treatment (EVT) techniques, specifically flow-diverting stents (FDSs), EVT has become a good option for these aneurysms. A literature review on EVT for paraophthalmic segment aneurysms is necessary. In this review, we discuss the anatomy of the paraophthalmic segment, classification of the paraophthalmic segment aneurysms, EVT principle and techniques, and prognosis and complications. EVT techniques for paraophthalmic segment aneurysms include coil embolization, FDSs, covered stents, and Woven EndoBridge devices. Currently, coiling embolization remains the best choice for ruptured paraophthalmic segment aneurysms, especially to avoid long-term antiplatelet therapy for young patients. Due to the excessive use of antiplatelet therapy, unruptured paraophthalmic segment aneurysms that are easy to coil should not be treated with FDS. FDS is appropriate for uncoilable or failed aneurysms. Other devices cannot act as the primary choice but can be useful auxiliary tools. Both coiling embolization and FDS deployment can result in a good prognosis for paraophthalmic segment aneurysms. The overall complication rate is low. Therefore, EVT offers promising treatments for paraophthalmic segment aneurysms. In addition, surgical clipping continues to be a good choice for paraophthalmic segment aneurysms in the endovascular era.

## Introduction

Traditionally, the ophthalmic segment of the internal carotid artery (ICA) has been defined as extending from the origin of the ophthalmic artery (OphA) to the origin of the posterior communicating artery (PcomA) (1). Recently, from an endovascular treatment (EVT) perspective, the paraophthalmic segment was proposed to optimize the description of the ICA originating from the distal border of the cavernous ICA and terminating at the PcomA origin (2). This nomenclature recognizes intrinsic uncertainty in the precise angiographic localization of aneurysms adjacent to the dural rings, regarding all lesions distal to the cavernous ICA as potentially intradural, which emphasizes their common features, and such lesions are increasingly addressed by endovascular means (2).

Aneurysms arising from paraophthalmic segments represent ~5–10% of intradural aneurysms (3, 4). Surgical clipping of these aneurysms poses challenges given complex nearby structures and a higher rate of visual complications (5, 6). Due to the advent of EVT techniques and devices, specifically flow-diverting stents (FDSs), EVT has become a good option for these aneurysms (7, 8). However, EVT may obviate some challenges. Currently, a complete review of the current status of EVT for aneurysms of the paraophthalmic segment is unavailable. Therefore, a review of the literature from a PubMed search is necessary. Additionally, we provide important images and educational cases in this review to increase reading interest.

## Anatomy of the paraophthalmic segment

**Figure 1:**
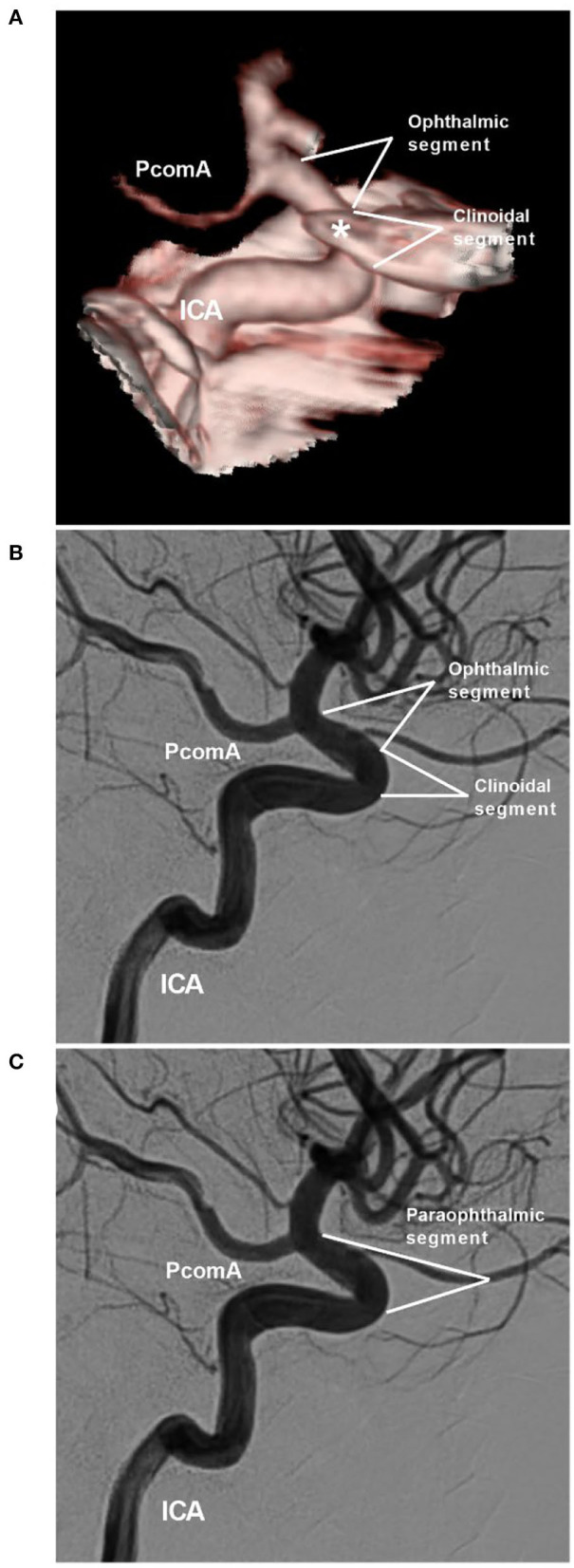
NYU paraophthalmic segment. **(A,B)** CTA **(A**) and DSA of the ICA **(B)** show the clinoidal and ophthalmic segments of the ICA. The segmentation is according to the classifications by Bouthillier and Ziyal. The asterisk in image **(A**) indicates the anterior clinoid process. **(C)** DSA shows the NYU segmentation. The clinoidal and ophthalmic segments of the ICA are combined into the paraophthalmic segment. CTA, computed tomography angiography; DSA, digital subtraction angiography; ICA, internal carotid artery; NYU, New York University; PcomA, posterior communicating artery.

The definition of the ophthalmic segment of the ICA is inaccurate because the OphA can arise from the low clinoid segment or high supraclinoid segment of the ICA (9). Shapiro et al. (2) proposed the influential NYU (New York University) segmentation system, which divides the ICA into seven segments: cervical, petrous, cavernous, paraophthalmic, PcomA, choroidal, and terminal segments. The paraophthalmic segment that originated from the distal border of the cavernous ICA incorporates the clinoid segments of the classifications of the ICA by Bouthillier and Ziyal (Figure 1) (10, 11). The clinoid segment is a short and variable-length portion of the ICA limited to the area between the distal dural ring and the proximal ring (carotid-oculomotor membrane) (9, 12).

Anatomically, the paraophthalmic segment of the ICA has two bends, of which the first is located at the ICA siphon where the vessel penetrates the dura, while the second is located near its termination; the length of this segment averages 1 cm (1). Regarding branches, the OphA is the largest branch of the paraophthalmic segment of the ICA; several large perforating vessels arise from the medial or inferomedial surface of the ICA and from the carotid cave of the ICA, and the number of perforators averages 3.6, of which the largest perforator is the superior hypophyseal artery (SHA) (1, 12).

## Classification of the aneurysms

Previously, according to their relationship to adjacent anatomical landmarks, aneurysms of the paraophthalmic segment of the ICA have had various names and types, such as clinoid, paraclinoid, supraclinoid, and ICA dorsal/ventral aneurysms (13). Therefore, a clear, unified classification has been necessary. In this review, peripheral-type aneurysms originating from the OphA were excluded because they are not located on the ICA (14). Traumatic aneurysms were also excluded because they do not share the same pathogenesis (15).

### Classification according to location

**Figure 2:**
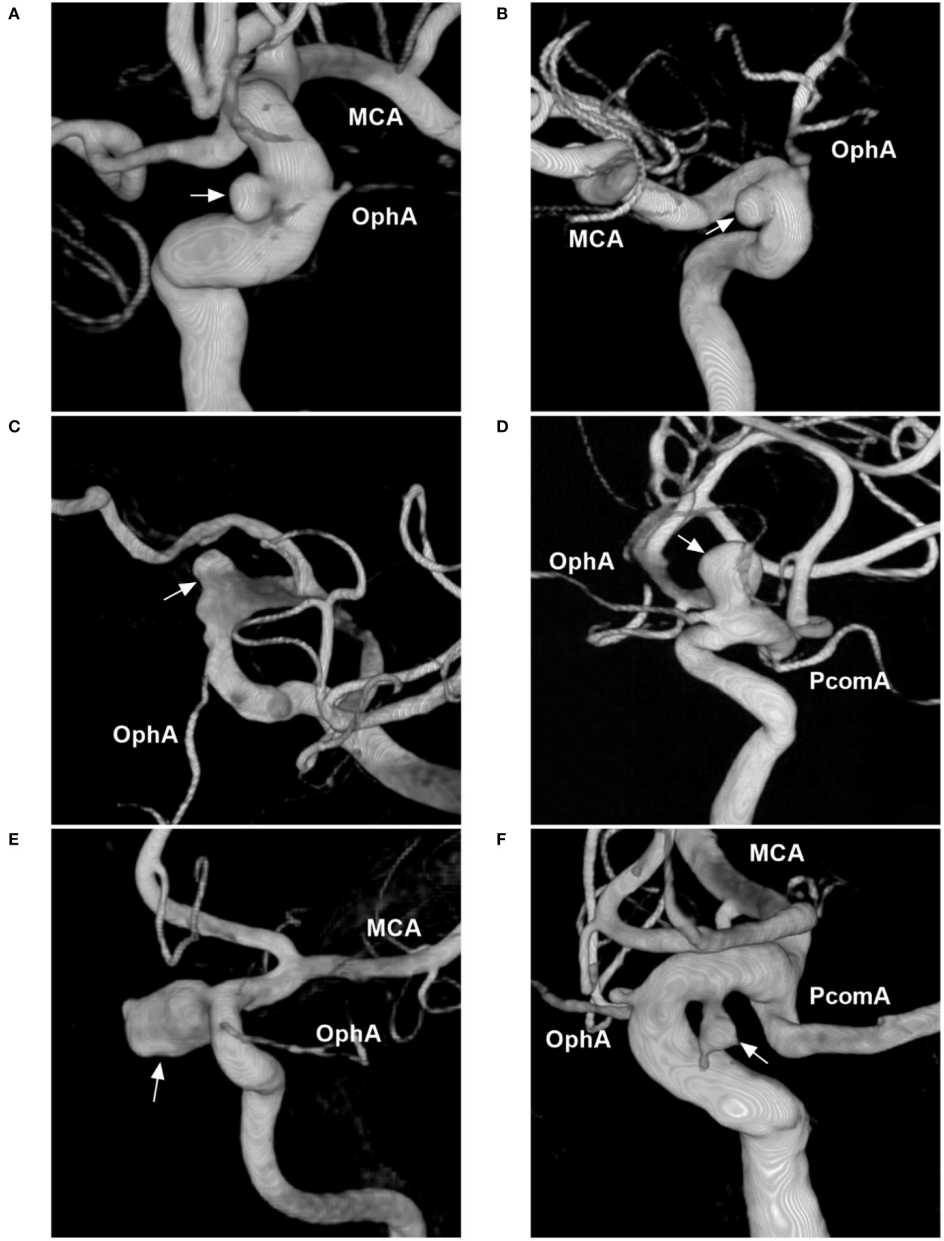
Classification of aneurysms of the paraophthalmic segment according to their locations. **(A)** Three-dimensional DSA shows a carotid cave aneurysm (arrow). **(B)** Three-dimensional DSA shows a clinoidal aneurysm (arrow) opposite the carotid cave. **(C)** Three-dimensional DSA shows an OphA aneurysm (arrow); the OphA was located away from the aneurysm neck. **(D)** Three-dimensional DSA shows an OphA aneurysm (arrow); the OphA arose from both the aneurysmal and ICA walls. **(E)** Three-dimensional DSA shows a suprasellar SHA aneurysm (arrow). **(F)** Three-dimensional DSA shows a paraclinoid SHA aneurysm (arrow). DSA, digital subtraction angiography; MCA, middle cerebral artery; OphA, ophthalmic artery; SHA, superior hypophyseal artery; PcomA, posterior communicating artery.

**Figure 3:**
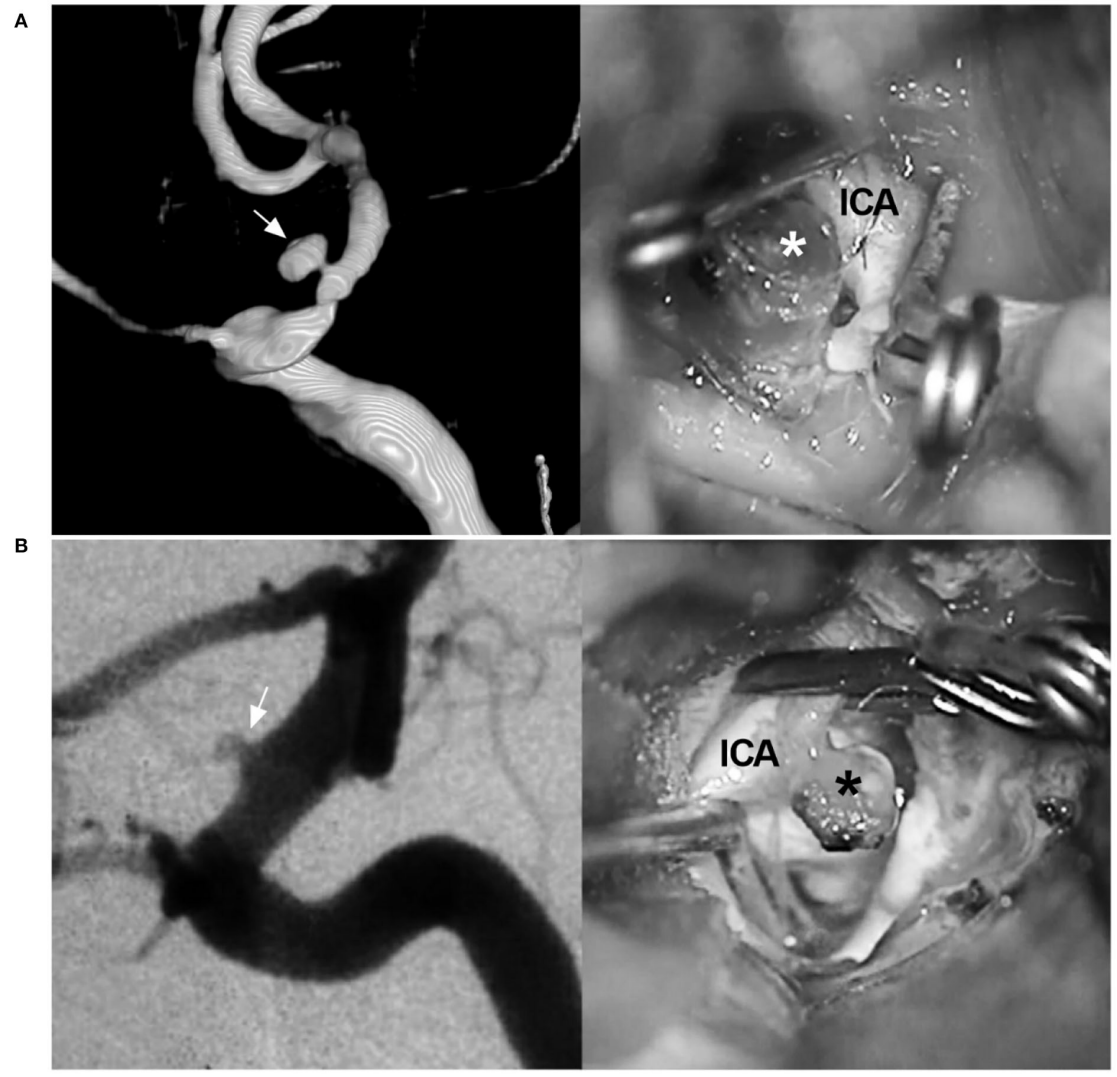
BBA and its differential diagnosis. **(A)** Left: Three-dimensional DSA of the ICA shows a mushroom-shaped BBA (arrow) on the paraophthalmic segment; Right: intraoperative image shows the fragile BBA without a definite neck (asterisk). **(B)** Left: DSA of the ICA shows an aneurysm (arrow) exactly like the BBA on the paraophthalmic segment; Right: intraoperative image shows that the aneurysm is saccular with a definite and stable neck (asterisk). BBA, blood blister-like aneurysm; DSA, digital subtraction angiography; ICA, internal carotid artery.

Aneurysms of the paraophthalmic segment are regarded as clearly or potentially intradural and include the categories of clinoid, carotid cave, OphA, and SHA aneurysms (2). Four classes can be proposed. The first type includes the clinoid and carotid cave families, which are totally or mainly located at the clinoid segment of the ICA (Figures 2A,B) (9, 16, 17). Carotid cave aneurysms belong to the clinoid segment and they share similar anatomic characteristics as clinoid aneurysms (9). However, carotid cave aneurysms are a unique subtype located in the carotid cave, which is a natural space medial to the clinoid segment of the ICA (12). The second type is OphA aneurysms (Figures 2C,D). The third type is SHA aneurysms (Figures 2E,F). The fourth type is the blood blister-like aneurysm (BBA), which originates at the perforator-free part of the anteromedial wall of the supraclinoid ICA (Figure 3) (4).

OphA aneurysms often arise from the ICA just distal to the OphA, pointing superiorly or superomedially. The locations of OphA aneurysms vary because although most OphAs originate from the intradural ICA, 2–8% of OphAs originate from the extradural ICA (18, 19). OphA aneurysms can be divided into separate and shared types (20). A separate-type aneurysm is defined as an OphA originating completely from the ICA wall, away from the aneurysmal neck (Figure 2C); in a shared-type aneurysm, the OphA originates from both the aneurysm and the ICA (Figure 2D). Separate-type OphA aneurysms are common (21).

SHA aneurysms arise from the ICA medial wall at the site of perforator origin, including suprasellar and paraclinoid variants (22). The suprasellar variant arises from the medial ICA wall and expands directly into the suprasellar space (Figure 2E). The paraclinoid variant arises from the inferomedial ICA and burrows down toward the carotid cave (Figure 2F). Because the origin of SHA may be in the carotid cave, when SHA aneurysms are large, they may involve the carotid cave (12, 23).

### Saccular, fusiform (dissecting), and blood blister-like types

**Figure 4:**
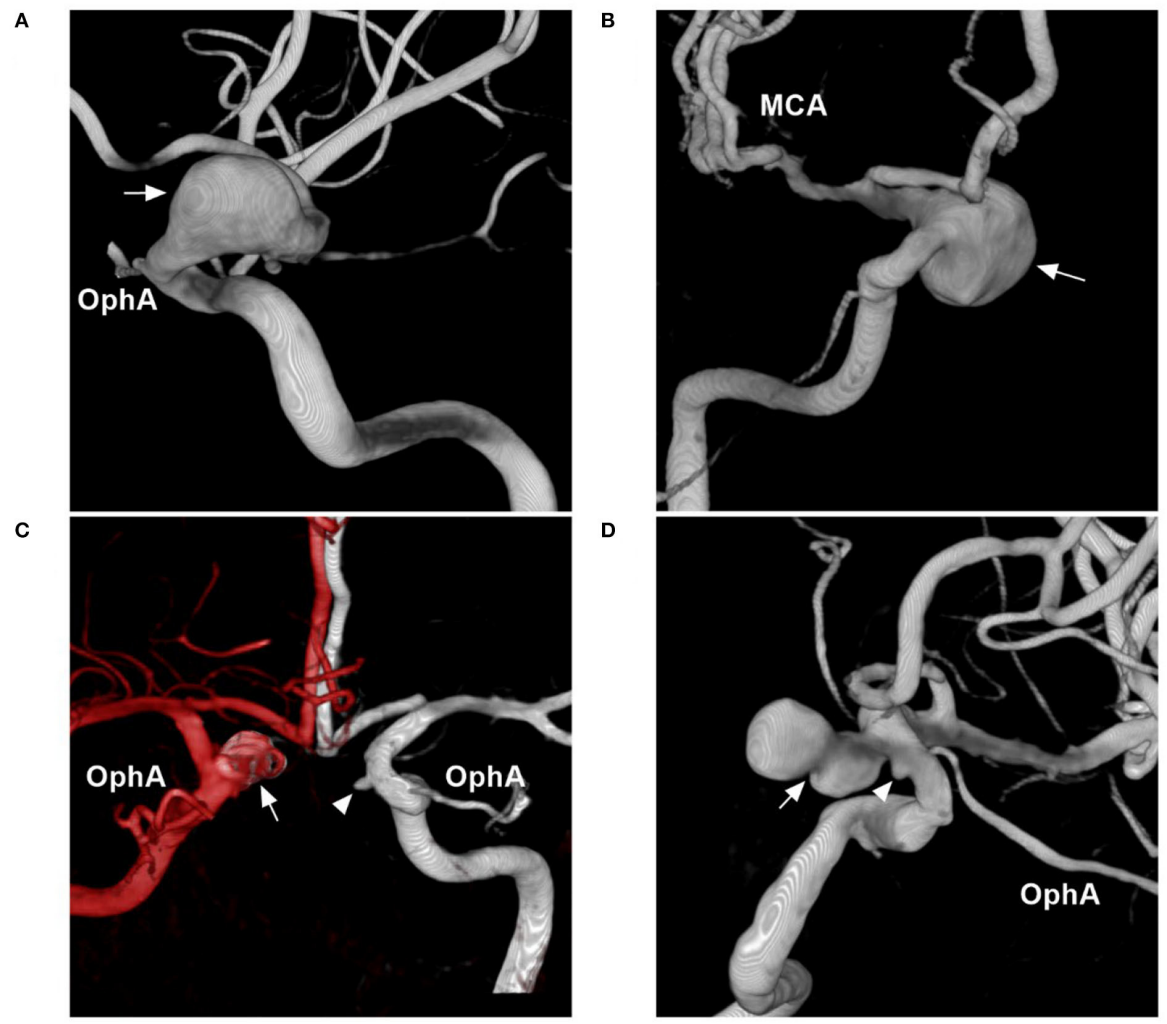
Other classifications of aneurysms of the paraophthalmic segment. **(A)** Three-dimensional DSA shows a fusiform aneurysm (arrow) of the paraophthalmic segment, which also belongs to dissection. **(B)** Three-dimensional DSA shows a large SHA aneurysm (arrow). **(C)** Three-dimensional DSA shows bilateral aneurysms: one is a coiled SHA aneurysm (arrow) and the other is a carotid cave aneurysm (arrowhead). **(D)** Three-dimensional DSA shows two tandem aneurysms: the large aneurysm is an SHA aneurysm (arrow), and the small aneurysm is a carotid cave aneurysm (arrowhead). DSA, Digital subtraction angiography; MCA, middle cerebral artery; OphA, ophthalmic artery; SHA, superior hypophyseal artery.

Based on their morphology and nature, aneurysms of the paraophthalmic segment can be divided into saccular, fusiform, and BBA types. Saccular aneurysms originate as branch-related aneurysms at the OphA and SHA. When the paraophthalmic segment is dilated, if the diameter of the dilatation is 1.5 times that of the normal ICA, fusiform aneurysms have formed, and they may be from dissection (Figure 4A) (24). Fusiform aneurysms often involve the long paraophthalmic segment and are called transitional aneurysms (25, 26).

In nature, BBAs are often located at the anteromedial wall of the supraclinoid ICA; they are false, consisting of a platelet plug covering a thin layer of adventitia overlying a defect in intima and media (27). BBAs have unique characteristics, such as hemispheric and broad-based appearances, and lack a neck. However, the identification of BBAs should be confirmed via microsurgery because some aneurysms that are initially indistinguishable from BBAs are not actually blood blister-like (Figure 3) (28, 29).

### Other classifications

Aneurysms of the paraophthalmic segment also have other classification systems, such as ruptured or unruptured; small (<7 mm), medium (7–12 mm), large (13–24 mm) (Figure 4B), or giant (>25 mm), and single or multiple (Figures 4C,D) (30–32).

## Principle and techniques of EVT

**Figure 5:**
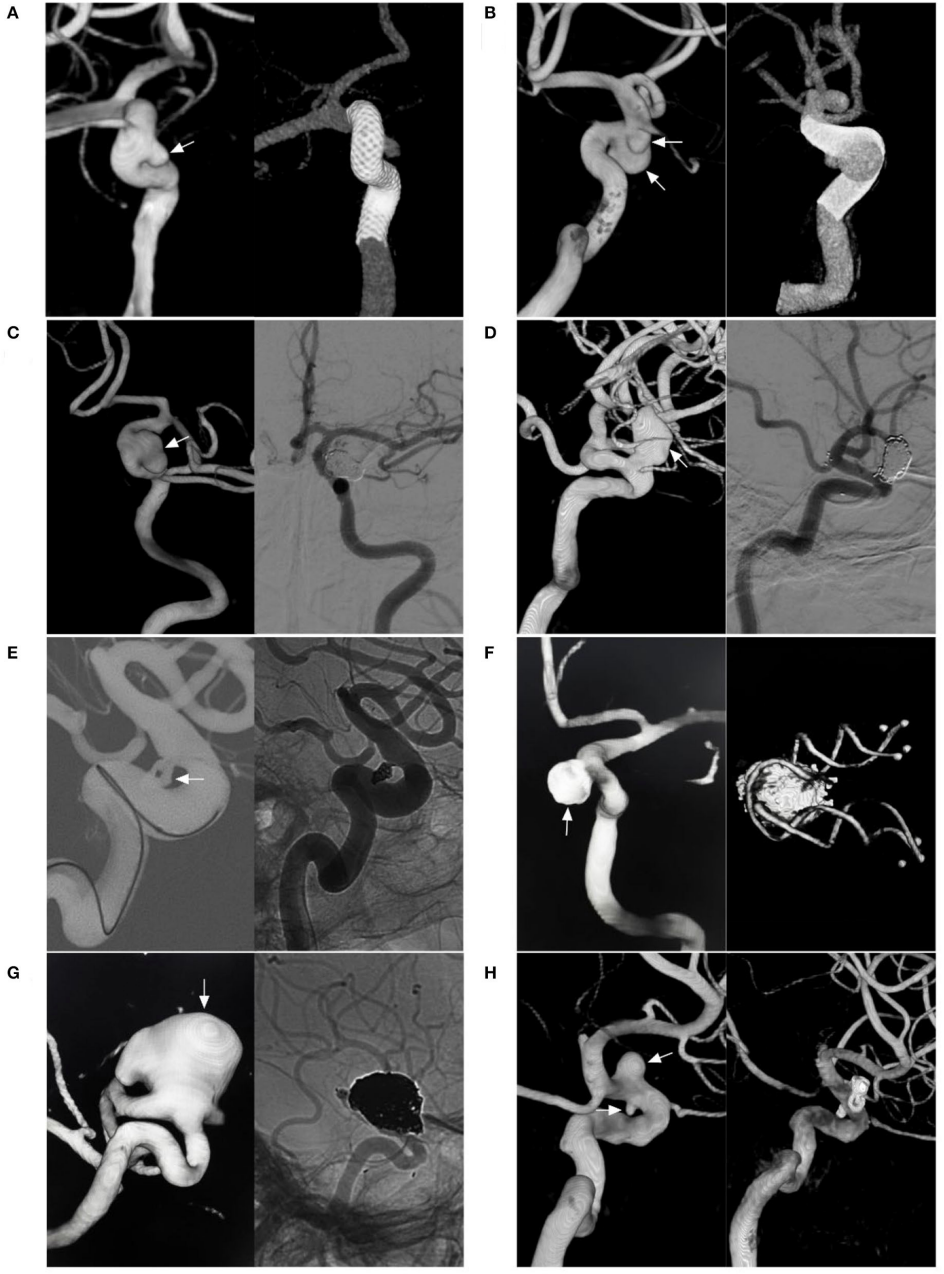
Various EVT techniques for aneurysms of the paraophthalmic segment. **(A)** Left, three-dimensional DSA shows a carotid artery aneurysm (arrow); right: Vaso CT shows the FDS covering the aneurysm. **(B)** Left, three-dimensional DSA shows two aneurysms (arrows) of the paraophthalmic segment; right: Vaso CT shows the FDS covering two aneurysms. **(C)** Left, three-dimensional DSA shows a clinoidal aneurysm (arrow); right: follow-up DSA shows that the aneurysms were coiled completely. **(D)** Left, three-dimensional DSA shows an OphA aneurysm (arrow); right: follow-up DSA shows that the aneurysm was coiled under stenting assistance. **(E)** Left, road map DSA shows a microcatheter into a paraclinoid-type SHA aneurysm (arrow); right: DSA shows the aneurysm was coiled. **(F)** Left, postoperative three-dimensional DSA shows a coiled suprasellar-type SHA aneurysm (arrow); right: Vaso CT shows the coils and stent. **(G)** Left, three-dimensional DSA shows a large OphA aneurysm (arrow); right: unsubtracted follow-up DSA shows that the aneurysm was coiled under stenting assistance. **(H)** Left, three-dimensional DSA shows OphA and SHA aneurysms (arrows); right: follow-up three-dimensional DSA shows that two aneurysms were coiled under stenting assistance. CT, computed tomography; DSA, digital subtraction angiography; EVT, endovascular treatment; FDS, flow-diverting stent; OphA, ophthalmic artery; SHA, superior hypophyseal artery; Vaso, vascular space occupancy.

EVT for aneurysms of the paraophthalmic segment includes the use of coil embolization, FDS, covered stents, and a Woven EndoBridge (WEB) device (MicroVention-Terumo, Aliso Viejo, California) (Figure 5). These aneurysms can also be treated with ICA occlusion, but FDS has eliminated the technique (33).

### EVT consideration based on natural history

Paraophthalmic segment aneurysms often grow slowly and are less likely to rupture than aneurysms in other categories. In a study by Jeon et al. (34) 524 patients harbored a total of 568 small unruptured paraclinoid aneurysms ( ≤ 5 mm). During the follow-up of 1675.5 aneurysm-years, the annual rupture rate and growth rates were 0.12 and 1.01%, respectively, and risk factors included lesions >4 mm in size, branch-related lesions, and multiple lesions. Close monitoring was only necessary for aneurysms with the above risk factors. Regarding the natural history of carotid cave aneurysms, Kalluri et al. reported 290 small (<4 mm) carotid cave aneurysms over 17 years, and no instances of aneurysm rupture or growth were found (35). Therefore, for these small carotid cave aneurysms, a watchful waiting strategy is feasible.

Therefore, EVT should only be indicated in large symptomatic aneurysms with mass effects on cranial nerves (36). In addition, due to aneurysms with regrowth or irregular shapes that confer a higher risk of rupture, EVT is necessary (37). Certainly, for ruptured aneurysms, EVT is mandatory (38–40).

### EVT consideration based on the collateral circulation of the OphA

Collateral circulation between external carotid artery (ECA) branches and OphA may be apparent or potential, varying from 36 to 89% of cases with ICA occlusion (41, 42). The balloon occlusion test (BOT) can identify collateral circulation (43). In the report by Kim et al. (42) on EVT for unruptured paraophthalmic segment aneurysms, after the BOT, intact collateral circulation was demonstrated in 92.9% of patients.

During the BOT, the ICA should be occluded, or the OphA orifice should be covered. HyperForm, HyperGlide (Medtronic Inc., Irvine, CA, USA), or Scepter balloons (MicroVention-Terumo, Aliso Viejo, California) can be chosen. After the balloon is inflated, contrast medium is injected into the common carotid artery. Positive BOT is defined as retrograde filling of the OphA with choroidoretinal blush. BOT may provide useful information to predict visual outcomes once OphA is threatened by EVT (42, 44). However, in OphA aneurysms with a positive BOT, intentional OphA occlusion should be avoided.

### Coiling with/without stent or balloon assistance

**Figure 6:**
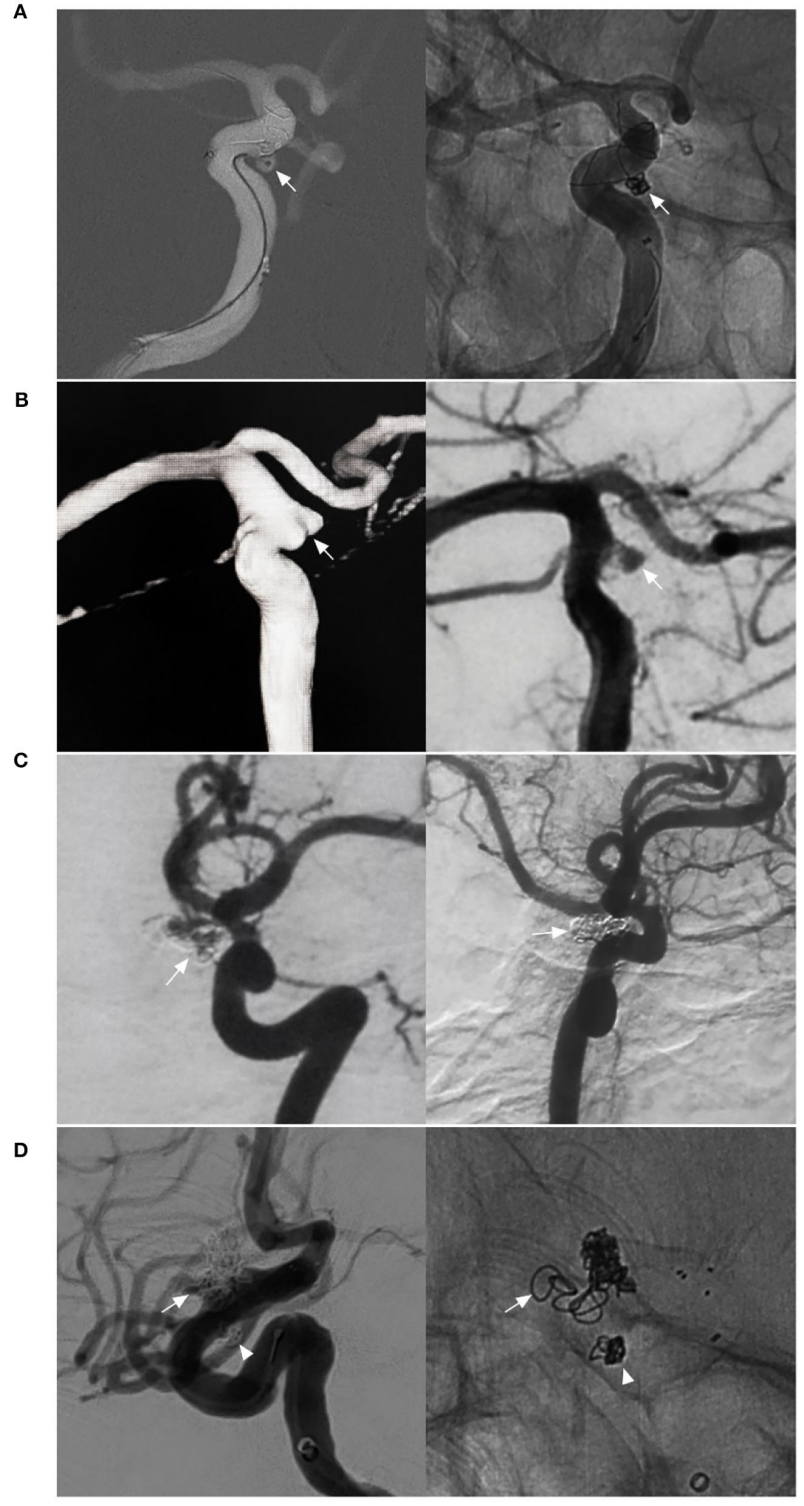
EVT difficulty in aneurysms of the paraophthalmic segment. **(A)** Left, Road map navigation shows the microcatheter tip (arrow) positioned in the aneurysm sac with the assistance of stent semi-deployment. The stent sealed a part of the aneurysm neck and provided the opportunity for the microcrater to enter the aneurysm; right: unsubtracted DSA shows the coils in the aneurysm (arrow). **(B)** Left, three-dimensional DSA shows a wide-necked lobulated carotid cave aneurysm (arrow); right: DSA shows the loose packing of the coiling in the aneurysm because the microcatheter was knocked off the aneurysm, but contrast agent retention can be seen in the aneurysm (arrow). **(C)** Left, DSA shows a recanalized aneurysm due to low-density packing (arrow); right: follow-up DSA shows the aneurysm had a complete embolization (arrow) after repeated coiling. **(D)** Left, DSA shows a recanalized lobulated upper aneurysm (arrow) and a completely embolized lower aneurysm (arrowhead); right: X-ray film shows that the upper aneurysm had low-density packing due to difficult catheterization (arrow), and the lower aneurysm had satisfactory embolization (arrowhead). DSA, Digital subtraction angiography; EVT, endovascular treatment.

For paraophthalmic segment aneurysms, the main difficulties in EVT are accurate positioning and stable support of the microcatheter (Figure 6). Due to the curvature of the ICA siphon and the aneurysms from the ICA sidewall, the sharp upturning of the microcatheter from the ICA to the aneurysm is difficult, stressful, risky, and sometimes impossible. The difficulty of catheterization is similar to that of EVT in the first segment of anterior cerebral artery aneurysms (45). For aneurysms of the carotid cave and clinoid on the sidewall of the ICA, the microcatheter is often most difficult to access for the aneurysm sac. The OphA and SHA aneurysms leave the ICA siphon and often have an upward or downward direction, and it is relatively easy for the microcatheter to access the aneurysms.

For access to aneurysms, microcatheter shaping is very important. The microcatheter shapes can be classified as straight, curved (45 and 90 degrees, J and C), pigtail (simple, right and left), and S-shaped (simple, right and left) (46). An S-shaped or straight microcatheter is helpful in superiorly directed aneurysms; a pigtail shape is useful in medially directed aneurysms (43). During microcatheter navigation, antegrade/retrograde shift, wire steering, looping, and coil or guidewire guidance can be used (46, 47). Despite these choices of microcatheter shaping, during coiling, the microcatheter tends to be knocked off the aneurysm, resulting in partial or low-density coil packing. In addition, due to OphA incorporation by aneurysms, efforts to save the OphA may result in incomplete coiling.

Single coiling can be used in narrow-necked aneurysms (31). However, for wide-necked aneurysms with a neck diameter > 4 mm or a dome-to-neck ratio <2, stent or balloon assistance is required (48). Old stents are challenging to deploy due to the acute curve of the ICA siphon; stents may kink or twist and have a defective wall attachment, which results in in-stent thrombosis (49). Currently, the low-profile Neuroform Atlas stent (Stryker Neurovascular, Fremont, California, USA) may be appropriate for deployment in the ICA siphon (50).

Coiling embolization with balloon assistance is a good choice due to the lack of antiplatelet therapy, which benefits ruptured aneurysms. HyperForm, HyperGlide, and Scepter balloons are useful. The balloon should be inflated while coiling. When finished, the microcatheter should be removed under balloon protection (51). Balloon inflation is limited to no more than 5 min at a time, alternating with at least 1 min of balloon deflation. In addition, a Scepter balloon can be used to release the Neuroform Atlas stent, which has additional advantages (52).

Regarding the need for antiplatelet use with stent-assisted coiling, a loading dose of oral aspirin (300 mg) and clopidogrel (300 mg) can be given at least 3 h before EVT for ruptured aneurysms, while a 3- to 5-day regimen of oral aspirin (100 mg/day) and clopidogrel (75 mg/day) is sufficient for unruptured aneurysms (50, 53). After EVT, dual antiplatelet therapy with oral aspirin (100 mg/day) and clopidogrel (75 mg/day) should be continued for 1–6 months depending on the stent (50). Dual antiplatelet therapy for 1 to 2 months is sufficient for the Neuroform Atlas stent due to low metal coverage, 3 to 6 months of dual antiplatelet therapy is necessary for the Solitaire stent (Medtronic, Irvine, California, USA) and Enterprise stent (Codman Neurovascular, Raynham, MA, USA), and 6 months of dual antiplatelet therapy is necessary for LVIS (MicroVention, Tustin, California, USA) and LEO stent (Balt Extrusion, Montmorency, France). Then, aspirin can continue to be administered daily at a dose of 100 mg for 3–6 months.

### FDS deployment

FDS can disrupt blood flow into aneurysms and act as a scaffold for endothelial cell proliferation (54). For aneurysms of the paraophthalmic segment, FDS is effective, as demonstrated by the Pipeline for Uncoilable or Failed Aneurysms trial (55). In addition, an FDS cannot be deployed to access the aneurysm sac, which reduces iatrogenic rupture from catheterization and coiling (21). However, FDS deployment in tortuous ICA siphons is not easy, and a good support system is important. After FDS deployment, the morphology and hemodynamics of the ICA siphon change, which may improve aneurysm healing (56). As technologies continue to evolve, FDSs will eventually become soft and pliable enough to deploy (57). FDSs will become easy to use in the ICA siphon.

**Figure 7:**
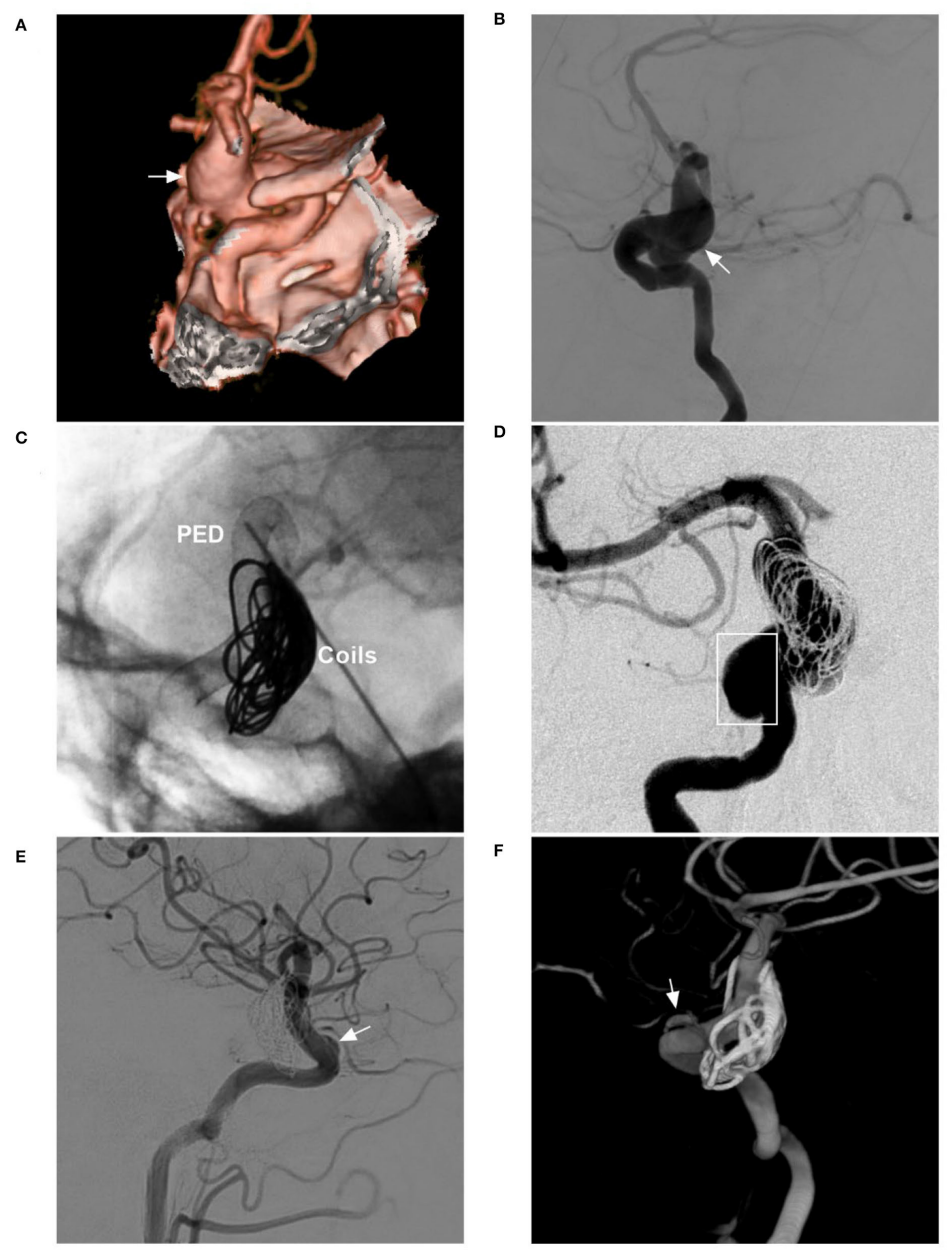
FDS deployment in a fusiform aneurysm under coiling assistance. **(A,B)** CTA **(A)** and DSA **(B)** show a large fusiform aneurysm (arrows) of the paraophthalmic segment. **(C)** X-ray shows the FDS deployment (PED) under the assistance of coiling (coils). **(D)** Postoperative DSA shows that the distal part of the aneurysm was coiled, and the proximal part (frame) was left. **(E,F)** Follow-up DSA at 6 months **(E)** and three-dimensional reconstructive DSA **(F)** show nearly complete occlusion; the arrows indicate the remnant neck. CTA, computed tomography angiography; DSA, digital subtraction angiography; FDS, flow-diverting stent; PED, pipeline embolization device.

**Figure 8:**
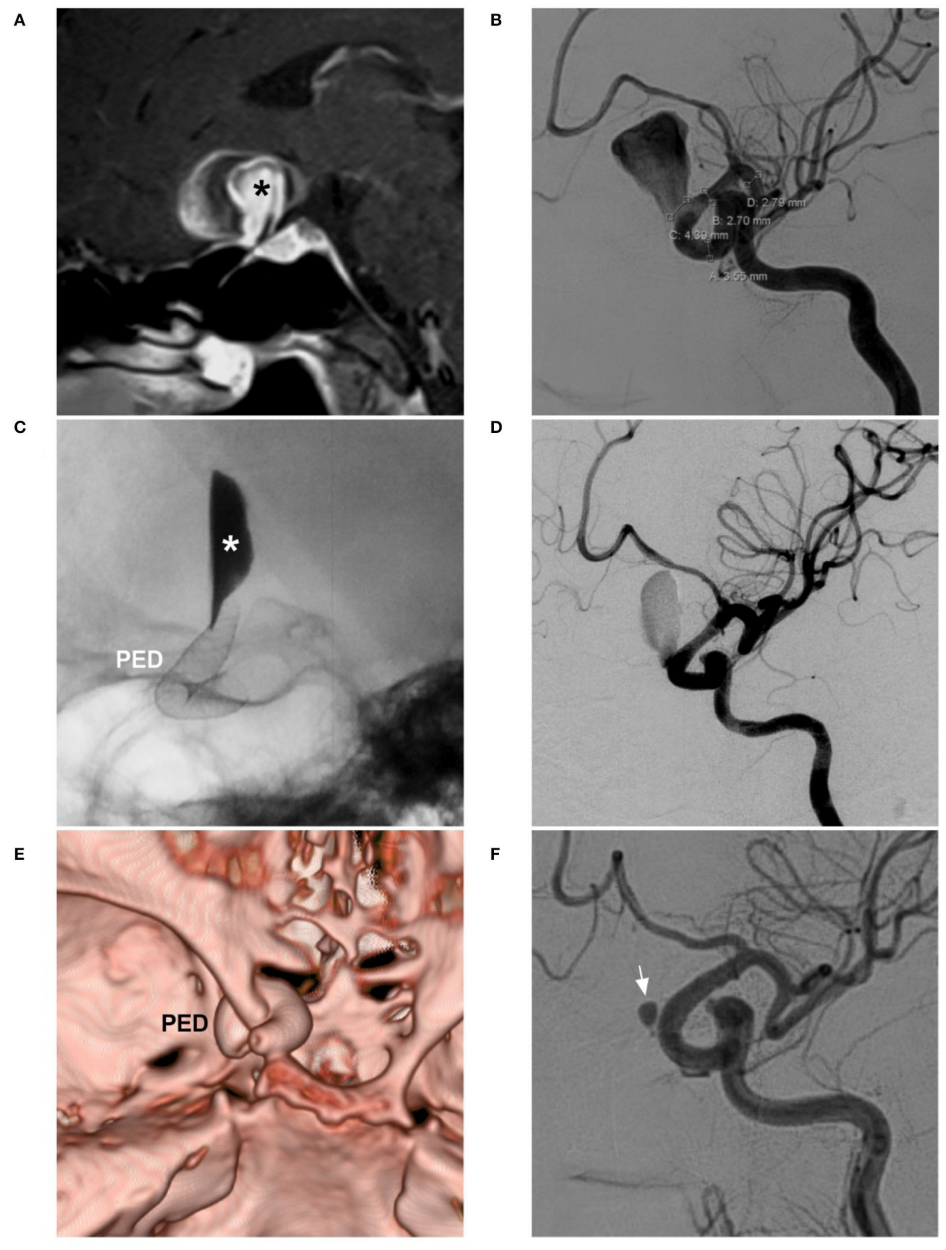
FDS deployment in a giant aneurysm without coiling. **(A)** Sagittal MRI shows a giant aneurysm with jet flow in the middle (asterisk). **(B)** DSA shows the aneurysm sac. **(C)** X-ray shows that contrast agent retention (asterisk) can be seen after FDS (PED) deployment. **(D)** Postoperative DSA shows reduced blood flow into the aneurysm. **(E)** Postoperative 1-week CTA shows the location of the PED. **(F)** Follow-up DSA at 6 months shows that most of the aneurysm was occluded and that complete occlusion occurred, and the arrow indicates the remnant part. CTA, computed tomography angiography; DSA, digital subtraction angiography; FDS, flow-diverting stent; MRI, magnetic resonance imaging; PED, pipeline embolization device.

For large fusiform aneurysms, adjunctive coiling can reduce FDS prolapse and act as a scaffold to organize thrombi, and the FDS should be supported by coiling until it is completely opened (Figure 7) (58). However, for giant aneurysms with mass effects, adjunctive coiling is not necessary because the optic nerve can be compromised by coiling via progressive mass effects or local inflammation (Figure 8) (59).

After FDS deployment, aneurysm healing is gradual. In a study of 44 patients with 46 unruptured aneurysms of the paraophthalmic segment by Burrows et al., the complete occlusion rates were 65, 78, and 96% at 6 months, 1 year, and 3 years, respectively (60). Therefore, FDSs do not result in immediate aneurysm closure for ruptured aneurysms. Ruptured aneurysms may not have sufficient time to wait for healing because the treatment effect is gradual. In fact, patients are still at risk of hemorrhaging after FDS deployment, and this is the main issue why separate, non–coiled-assisted FDSs are not suitable for acute cases (61). Due to the excessive use of antiplatelet therapy, unruptured paraophthalmic segment aneurysms that are easy to coil should not be treated with FDS (62).

Regarding the need for antiplatelet treatment with FDS deployment for unruptured aneurysms, patients should receive 100 mg of aspirin and 75 mg of clopidogrel daily for 5–7 days prior to the intervention (60). Platelet function assays should be performed to identify clopidogrel response. If the patient has been identified as a clopidogrel non-responder, ticagrelor may be selected instead. After EVT, dual antiplatelet therapy should be continued for 6 months; later, a daily dose of aspirin of 100 mg is used for life or at least for another half year.

### Covered stent placement

A covered stent can immediately lead to complete occlusion of aneurysms of the paraophthalmic segment (63). In the report by Yan et al. (64) of 49 intracranial aneurysms, 77.6% of aneurysms were large and located at the paraophthalmic segment; after covered stent (MicroPort, Shanghai, China) deployment, complete occlusion was achieved in 89.5% of aneurysms. However, stiff-covered stents have difficulty navigating through the ICA siphon, and procedure-related complications are non-negligible, including stent navigation failure, vasospasm, acute in-stent thrombosis, endoleak, and OphA, PcomA, or anterior choroidal artery occlusion (65, 66).

Regarding antiplatelet therapy, patients should be administered a preoperative double-antiplatelet regimen (100 mg aspirin and 75 mg clopidogrel) for at least 3 days (64). The postoperative double-antiplatelet regimen should continue for 6 months, and then a single-antiplatelet regimen (100 mg aspirin) should be continued for life (64). Currently, an FDS can replace a covered stent in most aneurysms of the paraophthalmic segment with fewer complications, especially when the side branches can be preserved (66).

### Woven EndoBridge (WEB) device

The WEB device is a self-expanding, retrievable, electrothermally detachable braided nitinol device that is placed within the aneurysm sac (67). It disrupts blood flow at the aneurysm neck and induces intra-aneurysmal thrombosis and offers a flat proximal surface that potentially supports the neoendothelium (68). In general, the WEB device in the aneurysm sac obviates the need for potent antiplatelet therapy if it does not protrude into the parent artery, which makes the WEB device appealing for ruptured and unruptured aneurysms (69).

The WEB device was initially designed to treat wide-necked bifurcation aneurysms. The use of the WEB device requires <45 degrees between the aneurysm and parent artery (70). Most aneurysms of the paraophthalmic segment are not appropriate for deploying the WEB device due to their directions. However, in selected aneurysms of the paraophthalmic segment, if the angle between the ICA and the aneurysm axil is <45 degrees and the proximal ICA near the aneurysm is not excessively tortuous, a WEB can be applied effectively (71).

### EVT for BBA of the paraophthalmic segment

For BBAs of the supraclinoid ICA, the optimal EVT has yet to be defined. Current EVT techniques include multiple overlapping stents with coiling, FDSs, and covered stents (72).

**Figure 9:**
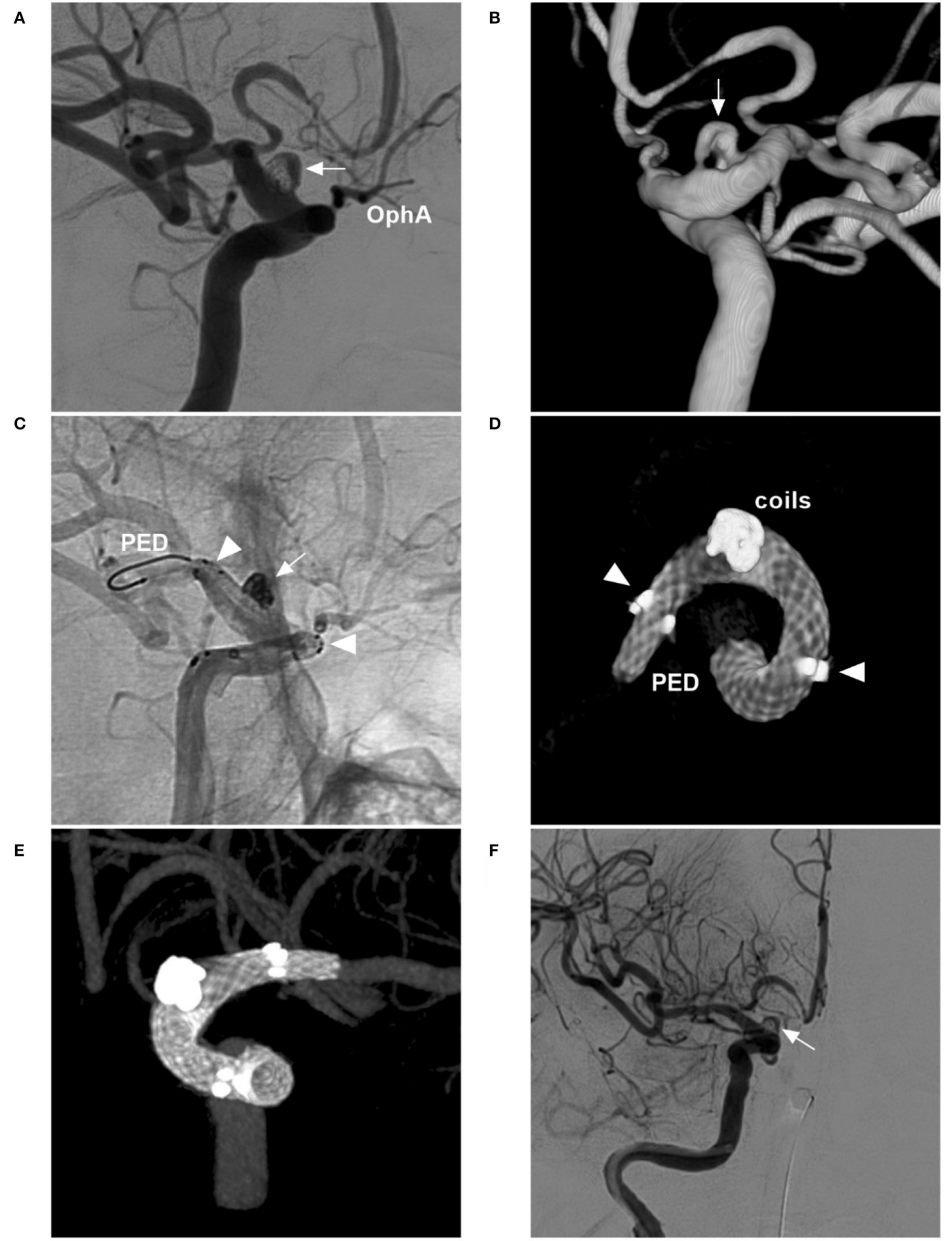
FDS deployment in a BBA with failed previous stent-assisted coiling. **(A,B)** Two-dimensional DSA **(A)** and three-dimensional DSA **(B)** show a BBA (arrows) with incomplete embolization of stent-assisted coiling. **(C)** Unsubtracted DSA shows the FDS (PED) crossing the BBA (arrow) and the FDS running in the stent (arrowheads). **(D,E)** Vaso CT without the vessel **(D)** and shows the FDS opened and covering the BBA in the stent (arrowheads). **(F)** Postoperative DSA shows the BBA without immediate complete occlusion; complete occlusion was expected by long-term follow-up. BBA, blister-like aneurysm; DSA, digital subtraction angiography; FDS, flow-diverting stent; PED, pipeline embolization device; Vaso, vascular space occupancy.

Stent-assisted coiling BBAs facilitate the placement of coils, but the complete occlusion rate is low. In a meta-analysis by Rouchaud et al. (73) of stent-assisted coiling BBA, complete occlusions were 33% initially and ~70% at mid- to long-term follow-up. FDS can be used to treat BBAs (Figure 9). FDS deployment for BBA of the supraclinoid ICA is also hindered by a low initial occlusion rate (36%) and its complication rate (17%), early rebleeding rate (7%), morbidity rate (13%), and mortality rate (9%) (73). Multilayer FDSs appear to be a promising strategy for BBAs, but sufficient evidence from trials is unavailable. A major disadvantage of FDS is dual antiplatelet therapy in the acute phase of ruptured BBAs (74).

In a recent meta-analysis by Lee et al. comparing stent-assisted coiling and FDS in the management of BBAs, the long-term complete occlusion rate was higher in the FDS group (89.3%) than in the stent-assisted coiling group (70.2%), and the rate of aneurysm recanalization was lower in the FDS group (4.5%) than in the stent-assisted coiling group (25.4%); however, the rates of mortality, favorable functional outcome, procedural complications, and rebleeding showed no differences between the two groups (75). Therefore, although FDS was associated with more favorable angiographic outcomes, FDS had similar complications and clinical outcomes as stent-assisted coiling, indicating little advantage in using FDS to improve BBA treatment.

In theory, covered stents can be used to treat BBAs of the supraclinoid ICA (76). However, due to the drawbacks mentioned above, especially intraoperative BBA rupture and endoleak, covered stents are not an ideal treatment for BBAs (77, 78). However, we look forward to advanced covered stents with good compliance, which will involve EVT for BBAs of the supraclinoid ICA.

In addition, the identification of true BBAs should be confirmed via microsurgery. Some putative BBAs of the supraclinoid ICA that were treated successfully by various EVT techniques in previous reports may not have been true BBAs but merely routine aneurysms (Figure 3) (79, 80).

## Prognosis and complications

For paraophthalmic segment aneurysms, the functional outcome is recorded using the modified Rankin Scale (mRS) score; aneurysm occlusion by coiling is categorized using the Raymond Roy Occlusion Classification; aneurysm occlusion of FDS deployment is categorized by angiography as complete, near complete (>90%), or incomplete (<90%) (81). Regarding the prognosis and complications, only coiling embolization and FDS deployment have been discussed, and other techniques are discussed in the corresponding sections.

### Prognosis

Both coiling embolization and FDS deployment can result in a good prognosis for paraophthalmic segment aneurysms. In a report by Adeeb et al. (81) good functional outcomes were 96.6 and 94.7% in-stent coiling and FDS, respectively. For paraophthalmic segment aneurysms with visual symptoms, coil embolization and FDS deployment can improve vision due to a reduction in mass effects and aneurysm pulsation (82, 83).

Regarding angiographic occlusion of the aneurysms, FDS deployment had a better outcome. In a meta-analysis by Touze et al. the complete occlusion rate for ophthalmic aneurysms after FDS deployment was 85% (54). For separate-type OphA aneurysms, the complete occlusion rate reached 89.5% (21). After the discontinuation of the second antiplatelet agent, the complete occlusion rate will increase (4).

Regarding coiling embolization, Wisniewski et al. reported that the overall recanalization rate ranged from 37.5 to 53% (4). Stent-assisted coiling lowers the recanalization rate; in long-term follow-up, the complete occlusion rate in-stent-assisted coiling may reach the rate of FDS deployment (84). In a report by Adeeb et al. on EVT for paraophthalmic segment aneurysms, the complete occlusion rate was 75.9% in-stent coiling compared with 81.1% in FDS deployment, and no significant difference was found (81).

### Complications

For EVT of paraophthalmic segment aneurysms, hemorrhagic and thromboembolic events, as well as visual deficits, must be considered (3, 85, 86). Aneurysm size influences complications; thromboembolic events are found more often in large aneurysms (7.4%) than in small ones (1%), whereas hemorrhagic complications are found in 0.7% of small aneurysms and no large aneurysms (53).

Thromboembolic events included in-stent thrombosis and distal cerebral ischemia (87). In the report by Di Maria et al., the overall rates in the coiling group and FDS group were 6.6 and 7.8%, respectively, and the rates of permanent morbidity in the coiling group and FDS group were 1.6 and 3.9%, respectively, and no significant difference was found between groups (88).

Regarding visual deficits, in coil embolization, the rate ranged from 4 to 10% (20). In FDS covering the OphA origin, the rate was 3% (54). One possibility is OphA occlusion with insufficient collateral circulation. The other is from thrombotic material, which is produced in aneurysms and migrates to the OphA (44). During EVT, acknowledging that tiny thrombi occlude the central retinal artery regardless of the collateral flow, intra-arterial injection of the glycoprotein IIb–IIIa inhibitor tirofiban can be helpful (89).

For large or giant paraophthalmic segment aneurysms, after FDS deployment, aneurysm volume can increase, resulting in a worse mass effect or delayed rupture due to thrombosis (24, 34, 88, 90). In addition, coiling embolization for large or giant aneurysms can be associated with the progression of mass effect and/or perianeurysmal inflammation, resulting in acute or delayed visual impairment (50, 91–93).

### OphA fate after FDS deployment

After FDS deployment, the blood flow of the OphA may be impeded or even occluded (94). In a report by Chalouhi et al. (95) with 95 patients whose OphA was covered by an FDS, during a follow-up of 7.5 months, OphA showed diminished flow in 4% of patients and occluded flow in 7% of patients; OphA occlusion was 8.6% when covered by one device vs. 21% when covered by two devices. Therefore, minimizing the number of FDSs across the OphA is a crucial factor to preserve its patency.

After FDS deployment, if the pressure gradient between the ICA and OphA is large and creates an aspiration effect that allows the continuation of blood flow, the OphA will always be patent, or the OphA will be occluded (96). In situations of robust collateral circulation, the OphA can obtain sufficient blood from the ECA, the pressure gradient may be abolished, and the OphA will be occluded (97). Due to the existence of sufficient collaterals, OphA occlusion usually has no overt consequences in the majority of cases (86).

## Surgical clipping

For paraophthalmic segment aneurysms, surgical clipping is still a therapeutic option and should always be considered (98). In a meta-analysis by Falk et al. (99) EVT and surgical clipping were compared, and no significant difference in clinical outcome was found between them for ruptured ophthalmic aneurysms. Aneurysms causing visual symptoms can be considered candidates for surgical clipping to improve visual function, and the recovery of visual function can often be expected when surgical clipping is performed rapidly before the visual dysfunction becomes irreversible (100). Moreover, surgical clipping is still a valuable option in younger patients to avoid long-term antiplatelet therapy (101).

## Summary

For paraophthalmic segment aneurysms, EVT techniques include coil embolization, FDSs, covered stents, and WEB devices. Coiling embolization remains the best choice for ruptured aneurysms. FDS is appropriate specifically for uncoilable or failed aneurysms. Due to the excessive use of antiplatelet therapy, aneurysms that are easy to coil should not be treated by FDS deployment. Both coiling embolization and FDS deployment can result in a good prognosis. For EVT of paraophthalmic segment aneurysms, the overall complication rate is low. Therefore, current EVT techniques offer promising treatment prospects for paraophthalmic segment aneurysms. Certainty surgical clipping continues to be a good choice in the endovascular era.

## Author contributions

JY contributed to the conception and design of the manuscript and critically revised the manuscript. YW wrote the manuscript and collected the medical records of the patients. Both authors approved the final version of this manuscript.

## Conflict of interest

The authors declare that the research was conducted in the absence of any commercial or financial relationships that could be construed as a potential conflict of interest.

## Publisher's note

All claims expressed in this article are solely those of the authors and do not necessarily represent those of their affiliated organizations, or those of the publisher, the editors and the reviewers. Any product that may be evaluated in this article, or claim that may be made by its manufacturer, is not guaranteed or endorsed by the publisher.
